# Induction mechanisms and strategies underlying interprophage competition during polylysogeny

**DOI:** 10.1371/journal.ppat.1011363

**Published:** 2023-05-18

**Authors:** Justin E. Silpe, Olivia P. Duddy, Bonnie L. Bassler

**Affiliations:** 1 Department of Molecular Biology, Princeton University, Princeton, New Jersey, United States of America; 2 Howard Hughes Medical Institute, Chevy Chase, Maryland, United States of America; Tufts Univ School of Medicine, UNITED STATES

## Introduction

Phages play central roles in shaping bacterial community biology. For example, lytic phages, by eliminating particular subpopulations of bacteria, control the composition of bacterial biofilm communities [1–3]. Temperate phages can infect and persist in bacteria, a state called lysogeny [4]. As inhabitants, lysogenic phages drive bacterial genome evolution via the introduction of viral genes that endow the hosts with new capabilities or that regulate host biochemical or signaling pathways. For instance, some cyanophages encode photosystem components that enhance host light harvesting ability [5,6]. In *Shigella flexneri*, phage-encoded enzymes modify O-antigen sugars enabling serotype conversion [7]. Similarly, phage enzymes hydrolyze the polysaccharide in the *Acinetobacter baumannii* capsule, altering biofilm formation [8]. Underscoring their importance to human health, temperate phages supply their bacterial hosts with the toxin-encoding genes responsible for diseases including cholera (caused by phage CTX of *Vibrio cholerae*), dysentery (caused by STX phages of *Escherichia coli)*, diphtheria (caused by phage beta of *Corynebacterium diphtheriae*), and botulism (caused by neurotoxin-encoding phages of *Clostridium botulinum*) [9–12]. These and other examples highlight fascinating connections between phage biology and bacterial biology. In some cases, phage infection confers benefits to the host bacterium, for example, enhancing colonization or dissemination from eukaryotic hosts. Phages are also frequently bacterial parasites, and, consequently, bacteria are under the pervasive threat of infection by phages that can exploit resources for continued propagation and, moreover, that can kill host bacteria in response to particular conditions.

**Fig 1:**
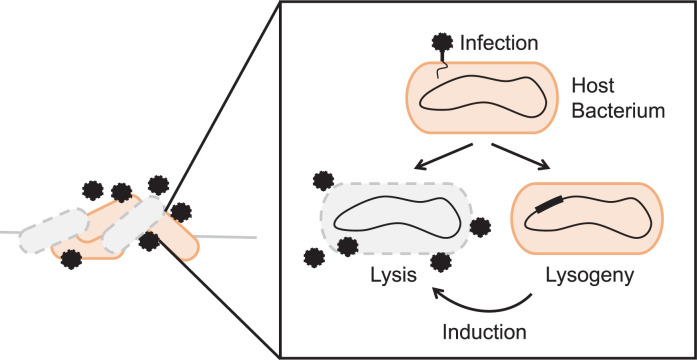
Prophage induction drives changes in bacterial communities. The cartoon shows possible effects of phage activity on a bacterial community. Black hexagons represent phage particles in the bacterial community. Orange and gray cells represent live and dead bacteria, respectively, the latter shown with hash symbols to denote disrupted membranes. Inset: The lysis–lysogeny switch is a phage-encoded regulatory decision-making program. Lysis leads to killing of the host bacterium and release of new phage particles. Lysogeny allows the phage to persist in the host cell and be passed down to progeny. The thick black region in the genome of the lysogenized bacterium represents the integrated phage genome. We note that, while not a main topic of this piece, phage infection in bacterial biofilms could be particularly interesting. Biofilms are surface-bound bacterial communities held together by an extracellular matrix. Biofilms can be composed of one or more bacterial species. Studies of prophage induction in bacterial biofilms show that, depending on the host ranges of the resident phages and the species composition of the bacteria present, induction of one prophage may kill the entire community or may target and eliminate specific bacterial species within the biofilm.

Following host infection, temperate phages undertake one of two lifestyle programs (Fig 1) [13,14]. They can enter the lytic cycle in which the phage uses host resources to replicate and package its genome into viral particles. The viral particles are subsequently released, killing the host cell and promoting phage dissemination to new host cells. Alternatively, temperate phages can enter into lysogeny and exist as prophages [13,14]. Commonly, the phage integrates its DNA at a discrete site in the host bacterial genome. Less commonly, the phage remains as an extrachromosomal element in a plasmid-like state [15,16]. In both cases, prophage replication during host cell division ensures transmission to progeny. Phages that enact lysogeny typically convert to the lytic route when the host bacterium experiences stress. The canonical trigger for the lysogeny-to-lysis transition is the activation of the bacterial SOS response following host DNA damage [13,14,17]. The notion is that by tuning into host SOS, the phage connects its lifestyle decision-making to host vitality. Phages thereby “abandon” their current hosts when host long-term survivability becomes uncertain.

Advances in high-throughput culturing, metagenomic sequencing, and genome assembly techniques are revealing new intricacies concerning the nature of phage–host and phage–phage interactions in real-world settings [18–23]. For example, most bacteria are predicted to harbor more than one prophage, a condition called polylysogeny [24–26]. Moreover, a growing number of phages isolated from different niches cannot be induced in the laboratory using standard induction conditions [27,28]. The majority of phage genes have no known functions [29], suggesting that, perhaps, some of these genes encode novel induction pathway components that enable multiple prophages to coexist and compete with one another. In this Pearl, we highlight newly discovered mechanisms underlying phage–phage competition with a focus on prophage induction.

## The canonical prophage induction cue: DNA damage

Extensive knowledge exists concerning molecular mechanisms underlying temperate lambdoid phages. Typically, entry into the lytic or lysogenic cycle is controlled by the master repressor of lysis, called cI [13]. cI-type proteins bind particular operator sequences in phage genomes where they function to repress transcription of lytic genes, driving lysogeny. Following host SOS induction, cI-type proteins are inactivated either by proteolysis, mediated by the host RecA protein, or by antirepression conferred by another phage protein called an antirepressor [14,30,31]. Consequently, lytic genes are derepressed, launch of the lytic cascade occurs, and the bacterial host cell is killed.

**Fig 2:**
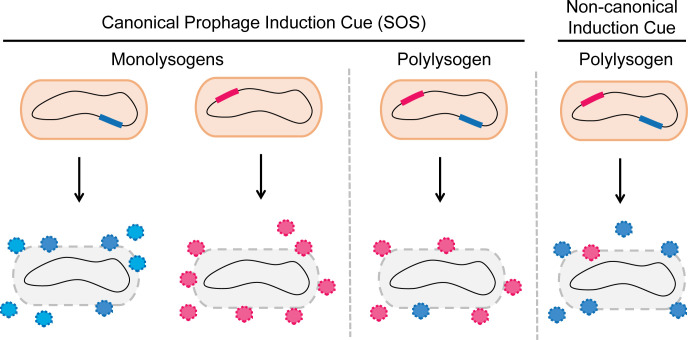
Phage productivity depends on the presence or absence of coinhabiting prophages and the mechanism of induction. Left: Two different prophages (blue and pink segments of DNA in the host genomes) inhabiting different bacterial cells (designated monolysogens). Viral particles may be produced in equal quantities (blue and pink particles) following induction by the canonical SOS trigger. Middle: If the two prophages inhabit the same bacterium (designated polylysogen), SOS induction leads to interprophage competition for host resources. Consequently, the phage that “wins” the competition (pink phage in the cartoon) will produce more virions than the other coresident phage (blue phage in the cartoon). Particles of the most competitive (pink) phage will primarily be released to spread within the bacterial community at the expense of the less competitive (blue) phage. Nonetheless, overall virion production is lowered in the polylysogen compared to the monolysogen. Right: In polylysogens, prophages that encode noncanonical induction pathways controlling their lysis–lysogeny programs (designated noncanonical induction cue) can outcompete coresiding prophages. This outcome occurs when the noncanonical induction cue is encountered. Only the phage that is responsive to the noncanonical induction cue (blue phage in the cartoon) replicates. In the examples in the figure, the pink phage “wins” the competition when the inducer is the canonical SOS cue, and the blue phage “wins” the competition when the inducer is the noncanonical cue. Thus, the particular induction cue dictates the outcome of the phage–phage competition.

Activation of the host SOS response is regarded as a “universal” inducer of prophages. Agents commonly used in the laboratory to drive prophage induction include DNA-damaging antibiotics and modulators of reactive oxygen species [32]. The discovery that most bacteria are polylysogens, coupled with the dogma that prophages are induced by host SOS, suggests that once the host cell is stressed, competition among phages would occur for appropriation of the host cell resources required for propagation [33,34]. Consequently, differences in replication rates, packaging rates, burst size, and other intrinsic phage properties that aid in the monopolization of host cell resources should dictate each phage’s success in producing progeny virions (Fig 2). Indeed, experiments show that only one temperate phage is predominantly recovered following SOS induction of a polylysogen, presumably the winner of the phage–phage competition [33,34]. Likewise in *E*. *coli*, in cases in which two lambdoid phages could be detected following induction, overall virion production was lower in the polylysogen than in a monolysogen [34]. Specifically, one phage exhibited its baseline productivity level while virion production by the other phage declined, or there was a loss in productivity of both phages [34]. These findings suggest that the host resources required for viral reproduction are limiting and/or interactions between prophage lytic programs cause mutual interference in the harnessing of host resources.

## Noncanonical prophage induction cues

A limitation of the above phage competition studies is that commonly used DNA-damaging agents fail to induce all prophages in the laboratory. Thus, DNA-damaging agents may not reflect the authentic cues that drive prophage induction in natural environments. Moreover, bacteria encounter environmental stressors that do not invoke the SOS response, suggesting that if prophage induction occurs under such conditions, induction must be triggered by SOS-independent cues. In support of this notion, accumulating evidence indicates that prophages have evolved to selectively tune into SOS-independent facets of host physiology; however, we note that such cues are generally orders of magnitude less potent at inducing the lysogeny-to-lysis transition than are SOS-dependent inducers. For example, salt stress and the intracellular ionic environment can bias phage λ^imm434^ toward lytic development, presumably because interactions between the λ^imm434^ cI repressor and its target operator DNA are sensitive to salt [35]. Administration of EDTA increases induction of lambdoid STX prophages [36]. The hypothesis is that divalent cation chelation disrupts the integrity of the bacterial outer membrane, which, by an unknown mechanism, functions as an inducing signal. Finally, overproduction of the RcsA or the DsrA small RNAs that control biofilm formation increases lambdoid prophage induction in *E*. *coli* [37–39]. Low temperature is an environmental stressor known to induce RcsA and DsrA, so their artificial overproduction likely mimics this condition [38]. The molecular mechanisms connecting the above prophage inducers to alterations in phage gene expression are mostly unknown. Also unknown is whether the stresses/mechanisms are highly specific for a single prophage or a single species of bacteria or, like DNA damage, are general.

Recent explorations into exotic prophage induction cues reveal that prophages surveil diverse aspects of bacterial physiology beyond host stress [40–45]. As one illustration, prophages detect bacterial-produced extracellular quorum-sensing (QS) signal molecules called autoinducers (AIs) [41–43]. QS is a chemical communication process bacteria use to synchronize collective behaviors [46]. Prophages harbor QS AI receptors, and detection of accumulated AIs launches the lytic cycle and release of viral particles. Thus, host killing occurs exclusively at high bacterial cell density when the probability of phage infection of another cell is maximized. For example, prophage ARM81ld of *Aeromonas* sp. ARM81 encodes a homolog of the bacterial LuxR-type QS receptor called LuxR_ARM81ld_ [41,47]. LuxR_ARM81ld_ detects its host *Aeromonas*-produced C4-homoserine lactone (HSL) AI and, in response, activates expression of a gene encoding an antirepressor [48]. The antirepressor inactivates the ARM81ld cI repressor, thus launching the phage lytic cascade. ARM81ld is also SOS inducible via proteolysis of its cI protein [48].

*Aeromonas* sp. ARM81 has recently been developed as a model polylysogenic bacterium as it harbors two prophages that employ distinct strategies for persistence. As noted, prophage ARM81ld is both SOS and QS inducible. The second *Aeromonas* sp. ARM81 prophage is called ARM81mr, and it is SOS but not QS inducible [48]. This arrangement makes it so that particular conditions could favor induction of one prophage over the other (Fig 2). Indeed, the ARM81mr prophage exhibits higher levels of spontaneous induction than does the ARM81ld prophage, and following DNA damage and SOS activation, higher levels of phage ARM81mr viral particles are released than are particles of phage ARM81ld [47,48]. By contrast, high levels of the C4-HSL QS AI induce the ARM81ld prophage but not the ARM81mr prophage, so almost exclusively phage ARM81ld viral particles are released [48]. Thus, whereas phage ARM81ld productivity is likely limited by competition with phage ARM81mr during SOS induction, selectively tuning into QS cues enables exclusive acquisition of host resources for phage ARM81ld propagation. In a fascinating twist, noncognate HSL AIs produced by non-*Aeromonas* bacteria repress LuxR_ARM81ld_ activity, thereby dampening ARM81ld prophage induction [43]. This feature could guard against premature launch of the prophage ARM81ld lytic cascade when the probability of encountering a suitable host for infection is low. Thus, particular environmental conditions apparently drive the outcomes of interprophage competition in polylysogens.

## Concluding remarks

Here, we considered interprophage competitive interactions during prophage induction in polylysogenic bacteria. Beyond the well-characterized SOS induction pathway, new studies are uncovering varied cues and physiological conditions that trigger prophage lytic programs and viral dissemination. Presumably, competition among prophages, together with relevant environmental cues, drives the evolution of their induction strategies. As results in this developing field of study continue to unfold, defining the molecular underpinnings of interprophage interactions, either competitive or cooperative, could be key to understanding how multiple prophages alter host biology in polylysogenic bacteria [49–51]. While not covered in this Pearl, cooperativity among prophages and prophage-like elements occurs and, indeed, can underpin host pathogenicity [31,52–54]. For instance, in *Staphylococcus aureus*, prophage-like mobile pathogenicity islands are repressed by a cI-type protein [55]. Their induction and, consequently, toxin production, occur in response to coresiding helper prophages that supply the needed antirepressor. Curiously, polylysogeny is widespread in bacterial pathogens, suggesting that the pathogenic lifestyle could select for acquisition and/or retention of multiple prophages. Given that virulence determinants are frequently encoded within prophages and can be produced during prophage induction, cross-regulatory prophage interactions could become promising targets for therapeutic intervention in clinically important bacterial pathogens.
